# Impact of Energy Drinks and Caffeine on Cognition, Stress, and Sleep During Examination: A Mixed-Methods Cross-Sectional Study From a Medical School in Mauritius

**DOI:** 10.7759/cureus.106320

**Published:** 2026-04-02

**Authors:** Krishna Dodia, Navita Jatain, Rudraksh Sharma, Shradha Banerjee, Sanjay Kumar Deshwali, Faraz Ahamed, Indrajit Banerjee

**Affiliations:** 1 Pharmacology, Sir Seewoosagur Ramgoolam Medical College, Belle Rive, MUS; 2 Research, Royal College Port Louis, Port Louis, MUS; 3 Biochemistry, Sir Seewoosagur Ramgoolam Medical College, Belle Rive, MUS; 4 Forensic Medicine and Toxicology, Sir Seewoosagur Ramgoolam Medical College, Belle Rive, MUS

**Keywords:** beverages, caffeine and addiction, caffeine consumption, caffeine consumption habits, coffee energy drinks, energy drinks, medical school students, sleep status, xanthines

## Abstract

Background: The consumption of caffeine-containing products such as coffee and energy drinks is highly prevalent among medical students, particularly during exams, due to their stimulating effects. The primary objective of the study was to assess the effect of energy drinks and caffeine consumption on cognitive performance, perceived stress, and sleep quality among medical students during examination periods in Mauritius.

Methods: An observational, cross-sectional, mixed-methods study was carried out at Sir Seewoosagur Ramgoolam Medical College, Mauritius, from November 12 to November 30, 2025, during the university examination phases. Quantitative data were collected via Google Forms (Google, Mountain View, CA) shared through the class WhatsApp group (Meta, Menlo Park, CA), while qualitative data were obtained through one-on-one interviews that were audio-recorded and manually transcribed. A convenience sampling technique was used to select the participants of the study. The data were entered and analyzed using SPSS version 31 (IBM Corp., Armonk, NY). The qualitative data were collected through one-on-one, face-to-face interviews conducted by the research team and were analyzed using NVivo 15 (Windows) software (QSR International, Melbourne, Australia). Braun and Clarke’s six-step thematic analysis method was used for data analysis. The quantitative study design and reporting were done according to the Strengthening the Reporting of Observational Studies in Epidemiology (STROBE) guidelines.

Results: A total of 69 students participated in the study, among whom 44 (63.8%) were females, and 25 (36.2%) were males. The mean age was found to be 21.67 ± 1.86 SD years. Consumption rates are high across all years, ranging from 65.5% to 84.2%. Higher daily intake (≥3 servings) is linked to a greater frequency of reported "caffeine crash" symptoms (55.6%), compared to moderate intake (one to two servings), where only 15.4% report such symptoms (p = 0.031). A higher percentage of caffeine consumers (42.0%) fall into the "High Stress" category compared to non-consumers (21.1%). Consuming caffeine after 6 pm is strongly associated with longer sleep onset latency (>30 minutes), reported by 64.0% of this group, compared to only 28.0% of those who stop consumption before 6 pm (p = 0 .009). A descriptive qualitative study was conducted with eight participants. Phenomenological thematic analysis was performed, which generated five themes as follows: academic influences, cognitive boosting, consumption patterns, sleep disruption, and risk-benefit perception. Each theme had different categories: exam pressure, perceived control, alertness, focus, productivity, and other variables.

Conclusion: During the examination period, caffeine usage was common among medical students across the years. Consuming caffeine after 6 pm was strongly linked to longer sleep latency, indicating that the timing of consumption influences sleep outcome. Overall, sleep quality is low, emphasizing their susceptibility to low-quality sleep during exams, and higher perceived stress was linked to changes in confidence levels.

## Introduction

In competitive academic environments, the consumption of caffeine-containing products such as coffee and energy drinks is highly prevalent among medical students, particularly during exams, due to their stimulating effects. Among the medical students in the United States, coffee was the primary source of caffeine consumption. Male students consumed an average of 53 mg/day caffeine from energy drinks compared to 30 mg/day in female students [1]. Medical students' consumption rate during exams is often higher than that of other students due to an extensive curriculum, prolonged study hours, fear of underperforming, and stress. The major reason behind consuming caffeine is its psychostimulant effect, as it acts as an antagonist to the adenosine receptors in the brain. According to a study conducted by Nehlig et al., moderate-dose caffeine has shown significant cognitive improvement, including increased alertness, memory retention, and reduced fatigue [2]. High doses of caffeine result in palpitations, anxiety, gastrointestinal symptoms, impaired motor functions, and a reduction in performance, which is known as caffeinism [3]. Caffeine intoxication is characterized by tachycardia, vomiting, cardiac arrhythmias, seizures, and, in severe cases, death [4].

Despite caffeine consumption, there is a greater preference for energy drinks among students due to their taste and caffeine concentration, which may be an amplified effect of caffeine on students [5]. Energy drinks are formulated with ingredients such as caffeine, taurine, vitamins, herbal supplements, and sugar [6]. Besides cognition, caffeine exhibits a well-documented impact on sleep. Energy drinks contain high concentrations of caffeine, and students tend to consume them during evening study sessions, which increases the latency of sleep and disturbs the sleep cycle, which is essential for cognitive recovery and memory [7]. According to a study conducted by Drake et al., a fixed dose of caffeine (400 mg) administered zero, three, and six hours before had a significant effect on sleep [7]. Caffeine’s effects on stress may vary depending on different factors [8]. To cope with a stressful academic situation, there is an upsurge in the consumption of caffeinated beverages among college students.

The need for the study arises from the fact that there is a dearth of information and a research gap regarding the impact of energy drinks and caffeine consumption on cognitive performance, perceived stress, and sleep quality among medical students during university examinations in Mauritius. The primary objective of the study was to assess the effect of energy drinks and caffeine consumption on cognitive performance, perceived stress, and sleep quality among medical students during examination periods at Sir Seewoosagur Ramgoolam Medical College in Mauritius.

## Materials and methods

Research design

An observational, cross-sectional, mixed-methods study was carried out at Sir Seewoosagur Ramgoolam Medical College, Mauritius, from November 12 to November 30, 2025, during the university examination phases. University professional phases are summative assessments undertaken by medical students. At Sir Seewoosagur Ramgoolam Medical College, which is affiliated to the University of Mauritius, there are four such phases: first professional, second professional, final part I, and final part II. A total of 69 medical students who were undertaking professional university examinations were included in the quantitative aspect of the study. In addition, a descriptive qualitative study was conducted in which eight participants, identified as P1-P8, were included; four of them were females, and four were males. Two students were selected from each professional phase (first professional, second professional, final part I, and final part II). A convenience sampling technique was used to select the participants of the study. The study design and reporting were done according to the Strengthening the Reporting of Observational Studies in Epidemiology (STROBE) guidelines [9].

Methodological triangulation

Methodological triangulation was used to enhance the credibility and robustness of the study. Quantitative data were collected alongside qualitative data from open-ended questions, which helped to explain the motivations underlying behaviors reported in the quantitative section. Both datasets were analyzed separately and then integrated to corroborate findings.

Methods of data acquisition

Quantitative data were collected via Google Forms (Google, Mountain View, CA) shared through the class WhatsApp group (Meta, Menlo Park, CA), while qualitative data were obtained through private, one-on-one interviews that were audio-recorded and manually transcribed. The interviews were conducted under the supervision of a senior author with no conflict of interest, ensuring participant anonymity and confidentiality. Informed consent was taken from all participants of the study.

Questionnaire design

After an extensive review of the literature, a structured questionnaire was developed. The questionnaire consisted of five sections for the quantitative domain and one section for the qualitative domain: (a) demographic and academic details, (b) caffeine and energy drink consumption patterns and dependence, (c) Perceived Stress Scale (PSS-10) [10], (d) sleep quality and study habits, and (e) self-rated cognitive performance and academic perception.

Validity and reliability of the questionnaire

The questionnaire was validated by five subject experts to ensure content, construct, and criterion validity. Each item was evaluated using a four-point Likert scale, and the average congruency percentage (ACP) was calculated following the method laid down by Polit and Beck [11]. Based on the experts’ feedback, necessary revisions were made, resulting in a final ACP of 90%. Additionally, a pilot study was conducted on five students to assess the face validity of the questionnaire. The PSS-10 is a well-validated tool used globally [10]. Cronbach's alpha was used to evaluate the reliability of the questionnaire (quantitative aspect). The internal consistency coefficient was found to be 0.812, indicating good and acceptable reliability of the questionnaire (Appendix).

Inclusion criteria

Medical students who were currently undertaking professional university examinations in various phases at Sir Seewoosagur Ramgoolam Medical College, Mauritius, were included in the study. Out of 251 students, 69 students completed and returned the Google Forms, which gives an overall response of 27.49%. A total of 69 students were included in the quantitative data analysis, while eight students, including four females and four males, identified as P1-P8, were included for qualitative data analysis. Two students were selected from each professional phase (first professional, second professional, final part I, and final part II).

Exclusion criteria

Students who were not taking part in the university examination were excluded. Students who did not provide consent to participate in the study and those who were taking medications that could affect sleep patterns were also excluded from the study.

Ethics approval

Ethical approval was obtained from the Institutional Research and Ethics Committee, Sir Seewoosagur Ramgoolam Medical College, Mauritius, on November 07, 2025 (Approval Code: SSRMC/IERB/2025/012) prior to the commencement of data collection. The study was conducted according to the latest version of the Declaration of Helsinki, which outlines the ethical principles for medical research involving human participants.

Sample size estimation

For the quantitative component, the sample size was calculated using the following formula:

$n=z^2 p(1-p)/d^2$

Where *n* is the required sample size, *z* represents the value of the standard normal distribution corresponding to 95% confidence interval, *p* is the estimated proportion in the population, and *d* is the absolute precision. Where *z* = 1.96 for 95% confidence interval, *p *= 0.5, and *d* = 0.122.

$n=1.96^2 X0.5(1-0.5)/0.122^2$

The required sample size was calculated to be 65. To compensate for non-responses, the sample size was increased by 5%, resulting in a final target of 68.25, which was rounded up to 69 participants to maintain adequate statistical power.

The data were collected during examination phases, and it was expected that students would be focused on their academic responsibilities and exam preparation, which would significantly reduce their availability and willingness to participate in study, resulting in an effective margin of error of 12%. Given the exploratory design, this sample size is acceptable for identifying preliminary patterns, though precision and generalizability are limited.

For the qualitative domain, data were collected in accordance with the guidelines of Glaser et al. and continued until data saturation was reached, indicated by the point at which no new codes/nodes emerged from the interviews [12].

Data handling and statistical methods

Quantitative Analysis

The data were entered and analyzed using SPSS version 31 (IBM Corp., Armonk, NY). Associations between categorical variables were analyzed using the chi-square test. For tables with expected cell counts less than 5, Fisher's exact test was employed, and p < 0.05 was considered indicative of statistical significance.

Qualitative Analysis

The qualitative data were collected through one-on-one, face-to-face interviews conducted by the research team. Each interview was audio recorded with participants' consent. Following the interviews, the recordings were manually transcribed verbatim, allowing careful examination and analysis of data. An inductive thematic analysis was conducted, allowing themes to emerge directly from the codes/nodes. Braun and Clarke’s (2006) six-step thematic analysis method was used for data analysis [13]. NVivo 15 (Windows) software (QSR International, Melbourne, Australia) was used for thematic phenomenological analysis to generate various nodes/codes and the various themes.

## Results

Quantitative analysis

A total of 69 students participated in the study, among whom 44 (63.8%) were females, and 25 (36.2%) were males. The mean age was 21.67 ± 1.86 SD years. Considering the participation and the examination undertaking, the majority were from the first professional phase (29, 42.03%), followed by the second professional phase (19, 27.54%), final part I (10, 14.49%), and final part II (11, 15.94%). No significant association was found between the year of study and whether a student consumes caffeine during exams (p = 0.606). Consumption rates were high across all years, ranging from 65.5% to 84.2%. All students diagnosed with a sleep or psychiatric disorder (n = 3) reported caffeine use. However, due to the small cell count, this association was not statistically significant (p = 0.551). This trend warrants cautious interpretation and further study (Table 1).

**Table 1:** Association between year of study, diagnosed sleep/psychiatric disorder, and caffeine consumption status. ^X^ P-value > 0.05 is non-significant.

Variable	Category	Caffeine consumers	Non-consumers	Total	Chi-square test/Fisher’s exact test	P-value
Year of study	First year	19 (65.5%)	10 (34.5%)	29 (100%)	χ² (3) = 1.84	0.606^X^
	Second year	16 (84.2%)	3 (15.8%)	19 (100%)		
	Third year	7 (70.0%)	3 (30.0%)	10 (100%)		
	Fourth year	8 (72.7%)	3 (27.3%)	11 (100%)		
Psychiatric disorder/diagnosed sleep disorders	Yes	3 (100.0%)	0 (0.0%)	3 (100%)	Fisher's exact test	0.551^X^
	No	47 (71.2%)	19 (28.8%)	66 (100%)		

Students primarily motivated by alertness or concentration are more likely to increase caffeine intake during exams (85.7%) compared to those motivated by habit, stress, or taste (63.6%), and a significant association exists (p = 0.039). Higher daily intake (≥3 servings) is linked to a greater frequency of reported "caffeine crash" symptoms (55.6%), compared to moderate intake (one to two servings), where only 15.4% report such symptoms, and a significant association is found (p = 0.031). Over half (58.0%) of caffeine consumers report at least sometimes feeling they need caffeine to function normally during exams, whereas no non-consumers reported this need; a highly significant association exists (p < 0.001) (Table 2).

**Table 2:** Patterns of caffeine consumption and dependence among medical students. * P-value <0.05 is statistically significant.

Variable	Category	Group 1	Group 2	Total	Chi-square test/Fisher’s exact test	P-value
Motivation versus changes	-	Alertness	Habit	-	χ² (1) 4.25	0.039*
	Significantly/Slightly increased	36 (85.7%)	14 (63.6%)	50 (78.1%)		
	Remained the same/Not regular	6 (14.3%)	8 (36.4%)	14 (21.9%)		
Servings versus crash	-	1-2 servings	>3 servings	-	Fisher’s exact test	0.031*
	Never/Rarely	33 (84.6%)	4 (44.4%)	37 (77.1%)		
	Sometimes/Often/Always	6 (15.4%)	5 (55.6%)	11 (22.9%)		
Consumption versus need to function	-	Consumers	Non-consumers	-	χ² (1) 20.01	<0.001*
	Never/Rarely	21 (42.0%)	19 (100.0%)	40 (58.0%)		
	Sometimes/Often/Always	29 (58.0%)	0 (0.0%)	29 (42.0%)		

While a higher percentage of caffeine consumers (42.0%) fall into the "High Stress" category compared to non-consumers (21.1%), this difference is not statistically significant (p = 0.108). The trend suggests a potential link between higher stress and caffeine use. Consuming caffeine after 6 pm is strongly associated with longer sleep onset latency (>30 minutes), reported by 64.0% of this group, compared to only 28.0% of those who stop consumption before 6 pm, and a significant association is present (p = 0.009). No significant association exists between daily study duration and self-rated sleep quality (p = 0.356). Poor sleep quality is prevalent (>65%) regardless of study hours, highlighting widespread sleep issues during exams. The vast majority (94.4%) of students who believe caffeine positively impacts their performance also rate their own cognitive performance during exams as "Same" or "Better." In contrast, a sizable minority (31.2%) of those who perceive no or negative impact from caffeine rate their own performance as "Worse." A significant association is found (p = 0.039). Students with high perceived stress are overwhelmingly (92.0%) clustered in the mid-range of confidence ("Slightly/Moderately confident"). Those with lower stress show a more polarized and varied confidence distribution, including more "Not at All" and "Very Confident" responses, and a significant association exists (p = 0.009) (Table 3).

**Table 3:** Association of caffeine use with stress, sleep, and cognitive performance during examinations. * P-value <0.05 is statistically significant. ^X^ P-value >0.05 is non-significant. PSS: Perceived Stress Scale.

Variable	Category	Group 1	Group 2	Total	Chi-square test/ Fisher’s exact test	P-value
Stress versus consumption	-	Consumers	Non-consumers	-	χ² (1) 2.58	0.108^X^
	Low stress (Score ≤ 19)	29 (58.0%)	15 (78.9%)	44 (63.8%)		
	High stress (Score ≥ 20)	21 (42.0%)	4 (21.1%)	25 (36.2%)		
Sleep latency	-	Last drink before 6 pm	Last drink 6 pm or later	-	χ² (1) 6.86	0.009*
	≤30 minutes	18 (72.0%)	9 (36.0%)	27 (54.0%)		
	>30 minutes	7 (28.0%)	16 (64.0%)	23 (46.0%)		
Sleep quality	-	Study < 7 hours/day	Study ≥ 7 hours/day	-	χ² (1) 0.85	0.356^X^
	Good (Very/Fairly good)	14 (40.0%)	10 (29.4%)	24 (34.8%)		
	Poor (Fairly/Very bad)	21 (60.0%)	24 (70.6%)	45 (65.2%)		
Cognitive performance	-	Positive impact	Negative impact	-	Fisher’s exact test	0.039*
	Same/Better	17 (94.4%)	22 (68.8%)	39 (78.0%)		
	Worse	1 (5.6%)	10 (31.2%)	11 (22.0%)		
Confidence vs. stress	-	Low stress (PSS ≤ 19)	High stress (PSS ≥ 20)	-	χ² (1) 6.90	0.009*
	Slightly/Moderately confident	28 (63.6%)	23 (92.0%)	51 (73.9%)		
	Not at all/Very confident	16 (36.4%)	2 (8.0%)	18 (26.1%)		

Qualitative analysis

A descriptive qualitative study was conducted in which eight participants labeled as P1-P8 were included; four of them were females, and four were males. Two students were selected from each professional phase (first professional, second professional, final part I, and final part II), and participants were purposively sampled to make sure balanced academic representation and equal gender distribution. All participants had previous experience with caffeine/energy drink consumption during examination periods. The interviews looked more at consumption patterns, perceived cognitive and academic impact, sleep-related impacts, psychological reliance, and risk-benefit perceptions.

Thematic analysis was performed, which generated five themes as follows: academic influences, cognitive boosting, consumption patterns, sleep disruption, and risk-benefit perception. Each theme had different categories: exam pressure, perceived control, alertness, focus, productivity, and other variables. Under each category, codes were organized as mentioned in Table 4.

**Table 4:** Qualitative thematic analysis.

Themes	Categories	Nodes/codes
Academic stress	Anxiety response	Increased nervousness
		Palpitation
		Restlessness
	Exam pressure	High stress levels
		Performance fear
		Syllabus overload
	Perceived control	Feeling productive
		Reduced fog
		Sense of control
	Psychological reliance	Confidence through caffeine
		Exam-specific dependence
		Performance association
Cognitive boost	Alertness	Increased vigilance
		Mental activation
		Reduced sleepiness
	Focus	Improved concentration
		Sustained attention
	Memory	Enhanced retention
		Improved memorization
	Productivity	Efficient revision
		Increased study hour
Consumption pattern	High intake	3-6 cups daily
		Large mug consumption
		Repeated refilling
	Preference	Black coffee
		Coffee-energy drink mix
		Limited energy drink use
	Situational use	Exam-only intake
		Morning boost
		Night-time use
	Tapering	Controlled withdrawal
		Dose reduction post-exams
		Gradual reduction
Sleep disruption	Poor quality	Light sleep
		Fragmented sleep
	Post-exam effects	Caffeine crash
		Withdrawal insomnia
	Reduced duration	All-nighters
		Early awakening
		Sleeping 3 hours
	Sleep onset issues	Delayed sleep
		Difficulty falling asleep
		Insomnia after intake
Risk-benefit perception	Perceived benefits	Academic support
		Necessary aid
		Productivity enhancer
	Perceived drawbacks	Anxiety increased
		Dehydration
		Energy drain

The different nodes/codes and narratives, along with their description, are mentioned in Table 5. The strongest themes in this analysis were palpitation, restlessness, increased nervousness, performance fear, syllabus overload, feeling productive, sense of control, increased vigilance, confidence through caffeine, enhanced retention, sustained attention, improved memory, mental activation, and various others.

**Table 5:** Codes/nodes generated.

Codes	Description	Narratives
Palpitation	Physical sensations of a rapid or pounding heartbeat attributed to caffeine.	“My caffeine intake increased a lot and I feel palpitations.” P3
Restlessness	Inability to relax, sit still, or remain calm after consumption.	“Excessive intake sometimes increases anxiety and restlessness.” P2
Increased nervousness	Heightened anxious feelings following caffeine intake.	“Caffeine definitely boosts anxiety and nervousness.” P4
Performance fear	Fear of poor grades, failure, disappointing expectations, or academic underperformance.	“Stress levels are quite high during exams due to syllabus and pressure to perform well.” P1
Syllabus overload	Statements indicating perception of excessive academic content, large volume of material, or insufficient preparation time.	“I usually study for longer hours, try to cover a large portion.” P5
Feeling productive	Perception of being efficient, effective, or task-oriented in studying.	“Caffeine helps me stay alert and more productive than I am.” P5
Sense of control	Feeling mentally organized, structured, or in command of academic tasks after caffeine intake.	“I make a schedule and work according to it and when I fix like a time for a topic, I’ve coffee and get through it and then move on to another topic.” P4
Confidence through caffeine	Belief that caffeine enhances self-confidence during exams.	“If I won’t have coffee, I won't be able to complete a single topic, that’s what I feel, and it helps me every time in exam.” P4
Exam-specific dependence	Reliance on caffeine, particularly during examination periods, rather than routine use.	“I am not that dependent on caffeine, but I do rely on it during my exam days.” P1
Increased vigilance	Heightened state of wakefulness and awareness.	“I had lots of syllabus to cover needed to stay awake and focused.” P6
Mental activation	Feeling mentally stimulated or cognitively energized.	“I have caffeine and it just boosts my mind.” P4
Sustained attention	Ability to maintain focus for longer, uninterrupted periods.	“Caffeine improves my attention span, can focus better.” P2
Enhanced retention	Improved ability to retain learned information.	“It is nice for recalling the prioritized high yield topics.” P2
Increased study hours	Extending the duration of study time due to caffeine.	“When I drink caffeine I feel motivated to study and can sit for longer hours.” P4
Morning boost	Use primarily to start the day or overcome morning fatigue.	“Caffeine intake generally during early morning sessions.” P2
Repeated refilling	Frequent re- consumption within short time frames	“I’m drinking as I finish my cup of coffee, I drink it again and make it again.” P2
Exam only intake	Caffeine consumption specifically during exams.	“Caffeine addiction only during exams I consume a lot and rest I never.” P4
Dose-reduction post exams	Lower intake once exam stress subsides.	“I do take caffeine, but in very small amounts after exams are done.” P2
Controlled withdrawal	Intentional reduction in caffeine after exams.	“I do not just stop taking caffeine, I tend to make it go slow.” P2
Light sleep	Superficial sleep with reduced depth or restfulness.	“It makes my sleep very light.” P4
Caffeine crash	Sudden fatigue or energy drop after the stimulant effect fades.	“Did not take caffeine, it would cause a lot of headaches.” P6
Withdrawal insomnia	Difficulty sleeping after stopping caffeine.	“I didn’t fall asleep for like 15 days, used to wake at 6 am due to caffeine.” P5
Delayed sleep	Taking longer than usual to fall asleep.	“It delays the sleep and reduces sleep depth.” P2
Insomnia after intake	Sleep disturbance directly following caffeine use.	“It really affects my sleep cycle.” P5
All-nighters	Staying awake the entire night to study	“I study for long hours, and I stay awake during the night.” P3
Academic support	Viewing caffeine as helpful in exam preparation.	“The benefits are something that can be counted upon and plays supportive role.” P5
Necessary aid	Belief that caffeine is required to cope academically.	“I believe the consumption is the key during the examination.” P2
Productivity enhancer	Perception that caffeine increases efficiency and output.	“Small amount of caffeine just helps me and enhance my productivity.” P1
Dehydration	Physical side effects such as dry mouth or thirst.	“Caffeine basically dehydrates me.” P5
Energy drain	Post-stimulant fatigue or crash.	“I feel caffeine drains me a lot.” P5
Balanced consumption	Belief that caffeine is safe if used responsibly	“It is just a temporary boost nothing else.” P3

## Discussion

Quantitative domain

The study examines the impact of caffeine and energy drink consumption on all medical students who were undertaking university examinations. It considered parameters such as cognitive performance, productivity, stress levels, sleep quality, and duration. Analysis revealed that the consumption pattern was more common among medical students; it was performance-driven, linked with sleep latency, associated with crash symptoms, dependency, and the need to function efficiently.

Prevalence and normalization of caffeine use

Among all academic years, caffeine consumption turned out to be the most common habit, with no statistical difference or association between the intake and the year of study. It proved that caffeine intake is a necessity not only in higher clinical settings but also in early medical students’ lives. A similar study demonstrating increased levels of intake and high prevalence has been reported among university students. McIlvain et al. were successful in showing their findings, where more than half of the university population was consuming caffeine regularly, and the intake was at its peak during academically challenging periods [14]. Similarly, Mahoney et al. explained a similar pattern of caffeine consumption in their study among university students, which increased during the exam time periods, and it primarily revealed academic performance as a key motivator for intake [1]. Unlike any other studies, which mainly demonstrated caffeine consumption in clinical settings due to higher workload, our findings were relevant enough to show an equal adaptation across all years, and this explains the peer-driven increase in caffeine intake among university students as a necessity.

Motivation-driven escalation during examinations

Many students explain that caffeine intake helps boost their concentration. Therefore, there is a significant increase in consumption during examination time when compared to those who consume caffeine just for taste and habit. This revealed a significant association linked between motivation for alertness and increased caffeine intake during examinations (p = 0.039). This study matches the pharmacological evidence, which proves that caffeine intake helps enhance vigilance, reaction time, and sustained attention at moderate doses [15]. A study conducted by Glade explains that caffeine is a central nervous system stimulant that, with the help of an adenosine receptor antagonism, majorly improves cognitive alertness in an individual [15]. Dawkins et al. stated in their study that it is the psychological beliefs that help us determine perceived performance improvement after caffeine intake, and not the pharmacological actions [16]. Our findings reveal that students who consume caffeine as a necessary aid were significantly more likely to report on the same or better performance, which supports the role of expectancy mechanisms alongside physiological stimulation.

Association between intake volume and "caffeine crash"

Along with caffeine intake, we commonly experience caffeine crash symptoms. There is a statistically significant link established between higher daily intake (three servings/day) and reported caffeine crash symptoms (p = 0.031). It was noted that more than 50% of the students experienced caffeine crash symptoms after high intake of caffeine when compared to moderate caffeine consumers, and this finding was supported by neurobiological evidence. Nehlig describes a rise in the upregulation of adenosine receptors due to repeated high caffeine intake exposure, rising sensitivity to rebound fatigue when stimulant levels reduce [2]. Juliano et al. explained the other side effects of high caffeine intake and stated the withdrawal-related fatigue, irritability, and reduced alertness in regular users, even after short abstinence periods [17]. In clinical settings, many studies have shown notable caffeine withdrawal, while some student-based studies reveal self-reported crash symptoms that were experienced during exam time. In comparison, our findings contribute behavioral evidence to the dose-dependent physiological response, highlighting potential risks of excessive stimulant reliance during academically stressful periods.

Dependence indicators and functional reliance

Talking about the population that consumes caffeine regularly on a daily basis, more than half of this population states that to function normally, caffeine is a must-have, whereas non-consumers have no such views. This showed a significant association between caffeine consumption and perceived “need to function” (p = <0.001). This pattern is consistent with developed evidence of caffeine dependency. Juliano and Griffiths talked about tolerance, withdrawal symptoms, and the constant desire to decrease fatigue as parameters consistent with mild substance dependence [17]. There is no evidence that shows disorder caused by caffeine use is universally classified as a clinical diagnosis, but the Diagnostic and Statistical Manual of Mental Disorders, 5th Edition (DSM-5) identifies caffeine withdrawal as a valid clinical entity. Our findings state that university examination pressure may lead to dependency, which further strengthens regular intake patterns. The absence of need among non-consumers makes the specificity of this association stronger and suggests that exam-related pressure may cause dependence-like behavior in an individual.

Sleep latency and timing of caffeine intake

There was a significant association noted between caffeine consumption after 6 pm and prolonged sleep onset latency (>30 minutes) (p = 0.009). All the students undertaking examinations tend to consume caffeine after 6 pm in the evening to stay alert and awake. Of these students, 64% faced difficulty falling asleep at their normal schedule (delayed sleep onset), while 28% of them did not face such problems due to early stoppage of caffeine consumption in the evening. A study by Drake et al. explains that consuming caffeine six hours before going to bed can significantly improve delayed sleep onset and decrease total sleep duration as well [7]. Our findings suggest that the real-world relevance of this association is evident, unlike other clinical scenarios, where findings show behavior during examination stress. Our study also provides an outlook where a student unknowingly perpetuates a cycle of caffeine consumption due to fatigue caused by delayed sleep onset and delayed sleep due to caffeine consumption, and the cycle repeats.

Sleep quality and study duration

There was no significant association between study hours and the sleep quality (p = 0.356), although reduced sleep quality was very common (>65%). This indicates that the timing of consumption and stimulants, along with stress, is more closely associated with disturbed sleep and not the study duration. Some studies suggest that academic and syllabus overload are the direct causes of poor and deficient sleep. However, our findings show that behavioral stimulant use plays a more crucial role during the examination period. This difference may indicate contextual dissimilarity between chronic academic overload and acute exam-related stress.

Stress and caffeine consumption

A greater number of students who consumed caffeine fell under the high-stress category, and the association was not statistically significant (p = 0.108). However, the pattern we observed suggests a potential relationship. A study by Rogers et al. explains that higher doses of caffeine consumption can lead to an increase in anxiety levels and related symptoms [18]. Whereas, compared with other studies, students who are finding it difficult to cope with high perceived stress tend to consume more caffeine than usual. This two-dimensional interaction may show the non-significant yet suggestive pattern experienced in our data. The absence of statistical significance reveals a limited sample size rather than the absence of an effect. Larger cohort studies may explain this relationship in a stronger manner.

Confidence and stress

The study demonstrated a significant association between stress levels and the confidence of students. It revealed that students who experienced very high perceived stress levels had moderate confidence levels. High stress levels are one of the main causes of reduced self-efficacy and poor confidence levels. This pattern may affect cognitive performance under stress, aligning with academic stress studies, which explains a decrease in performance confidence when under increased psychological stress.

Methodological triangulation was used to strengthen the study's credibility and robustness. Quantitative data, including demographic and academic details, caffeine and energy drink consumption patterns and dependence, PSS-10, sleep quality and study habits, and self-rated cognitive performance and academic perception, were complemented by qualitative data from open-ended questions, which provided insights into reasons behind underlying behaviors reported in the quantitative domains.

Qualitative domain

The qualitative part of the study shows that caffeine and its high intake are a necessary aid that helps students boost their mind and helps them cope with academic stress, increase their day-to-day productivity, elevate cognitive performance, and improve their ability to recall, focus, and stay alert, especially during examination time. However, many of them experienced drawbacks, including reduced sleep, anxiety symptoms, palpitations, restlessness, and caffeine crashes after peak use. The theme of academic stress was the most prominent among all the themes at Sir Seewoosagur Ramgoolam Medical College, and it was prevalent across all years of study, as it was stated as the reason why most students preferred consuming coffee. It was a belief that consuming caffeine would help them overcome the bulky syllabus, pressure, and fear of performing well.

We also found that the male population used caffeine to improve their performance, while the female population consumed caffeine more frequently to control their anxiety and calm themselves. As participant 4 stated in their interview, “I make a schedule and work according to it and when I fix like a time for a topic, I have coffee and get through it and then move on to another topic.” A study by Banerjee et al. explained that medical students often consume energy drinks due to exam stress and academic pressure, and they view caffeine as a practical way to handle their workload, not as something harmful [5].

The theme of cognitive boost elaborates on the belief of students that caffeine helps them increase their focus and alertness, helps them remember information for a longer time, and activates their mindset, as participant 2 shared, “caffeine improves my attention span, can help me focus better.” It improves their performance, which makes them feel productive, which further encourages them to keep using it. Other studies have reported similar findings: when medical students perceive that caffeine improves their thinking and studying ability, they tend to use it more over time. The theme of consumption patterns reveals that students often consume three to four servings per day, repeatedly refilling, and that intake is usually exam-dependent, with intake reducing as the exams are over. This study reveals that students usually adjust their caffeine consumption based on their academic needs, rather than drinking it habitually. As participant 2 described, “I do not just stop taking caffeine, I tend to make it go slow,” while participant 4 explained, “caffeine addiction only during exams, I consume a lot and rest I never.” This proved the pattern of consumption by students, which was nearly calculative and purely exam-dependent.

Another theme described as sleep disruption accounted for delayed sleep onset, light sleep, and broken sleep in students with caffeine consumption after evening. Many students described a cycle where they were trapped. They ingest caffeine at night to study, which delays sleep onset, then, because they sleep less, they feel very tired the next day, causing more consumption of caffeine. Participant 5’s experience of prolonged post-examination sleeping difficulty is consistent with DSM-5-recognized caffeine withdrawal syndrome, highlighting that the outcomes of examination-period caffeine use can persist even after the exam itself. These patterns are consistent with the quantitative evidence showing caffeine’s role in boosting alertness and cognitive processing following sleep loss, yet also disrupting sleep and contributing to anxiety, stress, and depression among students [3]. Our data uniquely provide relevant information about caffeine consumption and use as both a perceived academic aid and a contributor to psychological and sleep issues. It aligns with studies that link higher caffeine intake with perceived stress and poorer sleep among university populations.

Strengths and limitations

A key strength of the study is that the data were collected during the examination period, allowing for an adequate assessment of the effect of energy drinks and caffeine consumption on cognitive performance, stress, and sleep, while minimizing recall bias. To the best of our knowledge, this is the first study conducted and reported on the effect of energy drinks and caffeine among medical students during university examination phases in Mauritius. The main limitation of the study is the small sample size (quantitative domain) and a response rate of 29.49%, as the data were collected during the university examination time. During this time, students were focused on their academic commitments, which limited their availability and willingness to participate in the study. Although for the qualitative domain, an adequate sample was collected until data saturation was reached, indicated by the point at which no new codes/nodes emerged from the interviews.

## Conclusions

During the examination period, caffeine usage is common and is considered a normal behavioral trait among medical students, irrespective of the year of study. The findings of the study suggest that the primary motivating factor behind caffeine usage is to improve performance, and the desire for improved alertness and concentration usually leads to higher caffeine intake.

This suggests that there is an association between increasing daily caffeine intake and more severe symptoms of “caffeine crash.” Consuming caffeine after 6 pm was strongly linked to longer sleep latency and suggests that stimulant timings apart from duration are an important factor in determining sleep outcomes during examinations. The quality of sleep among this set of students was generally low, emphasizing their susceptibility to low-quality sleep during exams. Additionally, there was a significant link between high perceived stress and altered confidence distribution. Measures such as educational interventions focusing on sleep hygiene practices, stress management practices, and optimal performance enhancement strategies can be beneficial for reducing caffeine consumption.

## Appendices

**Table 6:** Questionnaire on the impact of energy drinks and caffeine consumption on cognitive performance, perceived stress, and sleep quality.

Section A: Demographic and academic details								
1	Age (years):							
2	Gender	Female	Male					
3	Year of study	1st year	2nd year	3rd year	4^th^ year			
4	Examination undertaking	First professional	Second professional	Final part 1 professional	Final part II professional			
5	Do you have any diagnosed sleep or psychiatric disorders?	Yes	No					
6	Do you take any medications that affect sleep, alertness, or concentration?	Yes	No					
Section B: Caffeine and energy drink consumption patterns and dependence								
1	Do you consume any caffeinated beverages (e.g., coffee, tea, soft drinks, or energy drinks) during the examination period?	Yes	No	If No, please proceed directly to Section C.				
2	Which of the following caffeinated products do you primarily consume during exams?	Coffee	Tea	Energy drink	Caffeinated sodas	Caffeine pills/tablets	Pre-workout supplements	Others
3	How has your consumption of these products changed during the current exam period compared to a regular academic week?	Significantly increased	Slightly increased	Remained the same	Slightly decreased	I do not consume them regularly		
4	On average, how many servings (cups/cans) of caffeinated beverages do you consume PER DAY during exams?	Less than 1	1-2	3-4	5 or more			
5	What is the primary source of your caffeine/energy drinks during exams?	Purchased from the college canteen/cafeteria	Purchased from off-campus stores	Prepared by myself (e.g., homemade coffee/tea)	Provided by friends/family			
6	What is your primary motivation for consumption?	To stay awake and alert for long study hours	To improve concentration and focus	To enhance memory/recall	To cope with stress or anxiety	Out of habit or for the taste	Social influence (friends/peers are also consuming)	
7	When do you typically consume your LAST caffeinated drink of the day during exams?	Before 4 pm	4 pm - 6 pm	6 pm - 8 pm	8 pm - 10 pm	After 10 pm		
8	After consuming caffeine or energy drinks, do you notice any of the following?	Increased alertness and energy	Improved mood	Jitters, anxiety, or restlessness	Heart palpitations or a fast heartbeat	Upset stomach or acidity	Difficulty falling asleep (when consumed in the evening)	No noticeable effects
9	Do you feel that you need caffeine or energy drinks to function normally or stay alert during exams?	Never	Rarely	Sometimes	Often	Always		
10	Do you experience "caffeine crash" (headaches, tiredness, irritability, brain fog) when you have not consumed caffeine for several hours?	Never	Rarely	Sometimes	Often	Always		
11	Have you ever tried to reduce your caffeine or energy drink intake but found it difficult?	No, I have not tried.	No, it was easy to reduce.	Yes, but it was only somewhat difficult.	Yes, it was very difficult.			
12	Do you continue to consume caffeine or energy drinks even when you know they cause you negative side effects (e.g., sleep disturbance, anxiety, jitters)?	Never	Sometimes	Often	I do not experience negative side effects.			
13	Would you describe yourself as dependent on caffeine or energy drinks?	No	Possibly	Yes				
Section C: Perceived Stress Scale (PSS-10)		0 = never	1 = almost never	2 = sometimes	3 = fairly often	4 = very often		
1	In the last month, how often have you been upset because of something that happened unexpectedly?	0	1	2	3	4		
2	In the last month, how often have you felt that you were unable to control the important things in your life?	0	1	2	3	4		
3	In the last month, how often have you felt nervous and "stressed"?	0	1	2	3	4		
4	In the last month, how often have you felt confident about your ability to handle your personal problems?	0	1	2	3	4		
5	In the last month, how often have you felt that things were going your way?	0	1	2	3	4		
6	In the last month, how often have you found that you could not cope with all the things that you had to do?	0	1	2	3	4		
7	In the last month, how often have you been able to control irritations in your life?	0	1	2	3	4		
8	In the last month, how often have you felt that you were on top of things?	0	1	2	3	4		
9	In the last month, how often have you been angered because of things that happened that were outside of your control?	0	1	2	3	4		
10	In the last month, how often have you felt difficulties were piling up so high that you could not overcome them?	0	1	2	3	4		
Section D: Sleep quality and study habits								
1	During the exam period, what time do you usually go to bed?	- (Hrs: Min)						
2	How long (in minutes) does it usually take you to fall asleep?	- Minutes						
3	What time do you usually wake up?	- (Hrs: Min)						
4	How many hours of actual sleep do you get on an average night during exams?	- Hours						
5	During the exam period, how often have you had trouble sleeping?	Cannot get to sleep within 30 minutes	Wake up in the middle of the night or early morning	Have to get up to use the bathroom	Cannot breathe comfortably	Feel too hot or too cold	Have bad dreams	Have pain
6	During the exam period, how would you rate your overall sleep quality?	Very good	Fairly good	Fairly bad	Very bad			
7	How often do you take daytime naps during exams?	Never	Rarely (1-2 times/week)	Sometimes (3-4 times/week)	Often (5 or more times/week)			
8	What is your typical daily study duration during exams?	Less than 4 hours	4-6 hours	7-9 hours	10 hours or more			
Section E: Self-rated cognitive performance and academic perception								
1	My attention and focus while studying are...	Much worse	Slightly worse	About the same	Slightly better	Much better		
2	My short-term memory and ability to recall studied material are...	Much worse	Slightly worse	About the same	Slightly better	Much better		
3	My mental alertness during lectures or study sessions is...	Much worse	Slightly worse	About the same	Slightly better	Much better		
4	My ability to concentrate for long hours is...	Much worse	Slightly worse	About the same	Slightly better	Much better		
5	Overall, how confident do you feel about your academic performance in the upcoming exams?	Not at all confident	Slightly confident	Moderately confident	Very confident	Extremely confident		
6	To what extent do you believe your caffeine/energy drink consumption impacts your exam performance?	Negatively impacts	No significant impact	Positively impacts	I do not consume these products.			
